# Potential Anticarcinogenic Peptides from Bovine Milk

**DOI:** 10.1155/2013/939804

**Published:** 2013-02-26

**Authors:** Giacomo Pepe, Gian Carlo Tenore, Raffaella Mastrocinque, Paola Stusio, Pietro Campiglia

**Affiliations:** ^1^Dipartimento di Scienze Farmaceutiche e Biomediche, Università degli Studi di Salerno, Via Ponte Don Melillo, 84084 Fisciano, Italy; ^2^Dipartimento di Chimica Farmaceutica e Tossicologica, Università di Napoli “Federico II,” Via D. Montesano 49, 80131 Napoli, Italy; ^3^Dipartimento di Biochimica e Biofisica, Seconda Università di Napoli, Via L. De Crecchio 7, 80138 Napoli, Italy

## Abstract

Bovine milk possesses a protein system constituted by two major families of proteins: caseins (insoluble) and whey proteins (soluble). Caseins (**α**_S1_, **α**_S2_, **β**, and **κ**) are the predominant phosphoproteins in the milk of ruminants, accounting for about 80% of total protein, while the whey proteins, representing approximately 20% of milk protein fraction, include **β**-lactoglobulin, **α**-lactalbumin, immunoglobulins, bovine serum albumin, bovine lactoferrin, and lactoperoxidase, together with other minor components. Different bioactivities have been associated with these proteins. In many cases, caseins and whey proteins act as precursors of bioactive peptides that are released, in the body, by enzymatic proteolysis during gastrointestinal digestion or during food processing. The biologically active peptides are of particular interest in food science and nutrition because they have been shown to play physiological roles, including opioid-like features, as well as immunomodulant, antihypertensive, antimicrobial, antiviral, and antioxidant activities. In recent years, research has focused its attention on the ability of these molecules to provide a prevention against the development of cancer. This paper presents an overview of antitumor activity of caseins and whey proteins and derived peptides.

## 1. Introduction

Milk proteins can exert a wide range of physiological activities, including enhancement of immune function, defense against pathogenic bacteria, viruses, and yeasts, and development of the gut and its functions [1]. Besides the biologically active proteins naturally occurring in milk, a variety of bioactive peptides are encrypted within the sequence of milk proteins that are released upon suitable hydrolysis of the precursor protein. A large range of bioactivities has been reported for milk protein components, with some showing more than one kind of biological activity [2]. Particularly, the present paper reviews the most important antitumor peptides derived from milk proteins (Table 1). 

Peptides derived from casein digestion have demonstrated antimutagenic properties [3]. Animal models for colon and mammary tumorigenesis have generally shown that whey protein is superior to other dietary proteins for suppression of tumour development [4]. This benefit is attributed to its high content of cystine/cysteine and *γ*-glutamylcyst(e)ine dipeptides, which are efficient substrates for the synthesis of glutathione, an ubiquitous cellular antioxidant that destroys reactive oxygen species and detoxifies carcinogens. Whey protein components, *β*-lactoglobulin, *α*-lactalbumin, and serum albumin were studied infrequently, but results suggest they have anticancer potential [2]. The minor component lactoferrin has received the most attention; it inhibits intestinal tumours and perhaps tumours at other sites. Lactoferrin acts by induction of apoptosis, inhibition of angiogenesis, and modulation of carcinogen metabolising enzymes and perhaps acts as an iron scavenger. Supplementing cows with selenium increases the content of selenoproteins in milk, which on isolation inhibited colon tumorigenesis in rats.

## 2. Caseins

### 2.1. *α*- and *β*-Casomorphins

*β*-Casomorphins (*β*-CMs) are a group of exogenous opioid-like peptides derived from the hydrolysis of *β*-casein and were first isolated from an enzymatic casein digest [6]. Their primary amino acid sequence is NH_2_-Tyr-Pro-Phe-Pro-Gly-Pro-Ile-Pro-Asn-Ser-Leu-COOH, located in bovine *β*-casein at positions 60–70. It was reported that *β*-CMs may reach significant level in the stomach because they are fairly resistant to proteolysis due to their proline-rich sequence [7].

*α*-Casomorphins (exorphins) have been isolated from peptic hydrolysates of *α*-casein fractions. In general, their structures differ considerably from those of *β*-casomorphins. Active fractions were shown to be a mixture of two separate peptides derived from *α*_1_-casein fragments 90–95 and 90–96 [Arg_90_-Tyr-Leu-Gly-Tyr-Leu_95_-(Glu_96_)]. The N-terminal arginine residue was also reported to be essential for opioid activity.

*α*- and *β*-Casomorphins may be produced by the enzymatic action of different proteases released from tumor cells [8, 9]. Indeed, Hatzoglou et al. [10] have shown that five different casomorphins, *α*-casein fragments 90–95 and 90–96 [11], *β*-Casomorphins 7 (BCM7) fragment 60–66, *β*-Casomorphins 5 (BCM5) fragment 60–64, and the morphiceptin, the amide of *β*-Casomorphins 4, have an antiproliferative action on T47D cells, blocking cells in G0/G1 phase. 

It is, therefore, possible to make two important considerations: one, on the inhibition that appears to be due to the interaction between the casomorphins and opioid receptors binding sites, decreasing cell proliferation in a dose-dependent manner; another, covering the essential requirements for the interaction of casomorphins with opioid receptors, that are identified by the hydrophobic character of proteins, their high content of proline and the presence of tyrosine at the N-terminal [12]. Also, it appears that, with the exception of morphiceptin whose action is mediated by type II somatostatin receptors, all peptides interact with to *δ*- and *κ*-opioid binding sites of T47D cells with different selectivity. It is interesting to note that all casomorphins show a significant interaction with somatostatin receptors in T47D cells, as these peptides may have a major physiological role in breast cancer. Furthermore, the interaction of casomorphins with opioid and somatostatin receptors leads to the inhibition of intracellular levels of cAMP.

Subsequently, Kampa et al. [13] have identified a new casomorphin pentapeptide (*α*_s1_-casomorphin), isolated from *α*_s1_-casein, with the sequence Tyr-Val-Pro-Phe-Pro (f158–162), capable of inhibiting, in a dose-dependent and reversible manner, the proliferation of T47D human breast cancer cells. Differently from other casomorphins, *α*_s1_-casomorphin does not interact with somatostatin receptors in our system.

Recently, De Simone et al. [14] have isolated a partially purified peptide subfraction from buffalo cheese acid whey, called f3, which reduces colon cell proliferation about 30% compared with the control sample represented by H-CaCo2 cells untreated with peptide extract.

The cytomodulatory and antioxidant effects of complex peptide fraction of the buffalo milk waste whey (BWW) in human epithelial colon cancer cells are due at a reduction of mitochondrial superoxide anion level and a subsequent decrease in Hsp 70 and 90 expression. Moreover, a 5-fold decrease was observed in cyclin A expression and cell cycle arrest in G1/G0 phases. These responses were associated with increases in expression of alkaline phosphatase activity, marker of enterocytic differentiation, and senescence-associated (*β*)-galactosidase.

Moreover, it seems that inhibition effects of f3 on H-CaCo2 cell proliferation may be mediated by secretion of ceramides which resulted in cell cycle arrest, differentiation and in subsequent accelerated senescence.

The structural analyses carried out on f3 showed the presence of peptides *β*-CN f57–68 and f60–68, precursors of the agonist opioids BCM7 and BCM5 [15]. These peptides could reduce colon cells proliferation by interaction with specific opioid, and somatostatin receptors, present in the intestinal tract of mammals.

### 2.2. Caseinphosphopeptides

Caseinphosphopeptides (CPPs) are a family of bioactive peptides derived from digestion of casein. Their name is due to their high content of phosphorylated sites, and they are characterized by the ability to bind and solubilize calcium [16]. This property is responsible for their anticancer activity against intestinal tumor HT-29 cells, by modulating cell proliferation and apoptosis.

More recently, it was demonstrated that in HT-29 cells, as well as in a primary human colon cancer cell line (AZ-97), the activation of voltage-activated L-type calcium channels, which mediate the calcium influx according to the depolarization state of the cell, is correlated to apoptosis, and their blockade may promote the growth of colon cancer cells [17]. Perego et al. [18] have demonstrated that CPPs protect differentiated intestinal cells from calcium overload toxicity, prevent their apoptosis favoring proliferation, and at the same time induce apoptosis in undifferentiated tumor cells. Probably, this effect is the result of binding of CPPs with extracellular calcium with a precise dose-response relationship, causing a reduction in the cell proliferation rate and apoptosis. In fact, antagonists of calcium channels abolish the response to CPPs or reduce both percentage of responsive cells and the increase of intracellular calcium concentration.

## 3. Whey Proteins

### 3.1. *α*-Lactoalbumin

*α*-Lactoalbumin (*α*-LA) is, quantitatively, the second most important protein in whey, and it contains 123 amino acid residues with a molecular weight of 14,175 kDa and isoelectric point between 4.2 and 4.5 [19]. In aqueous solution, *α*-LA has a globular structure stabilized by four disulphide bonds, and, actually, three genetic variants (A, B, and C) have already been identified [20]. This globular protein consists in a single polypeptide chain with eight cysteine residues, and it is physiologically important because of its requirements in lactose synthesis.

Some important bioactivities have been reported for *α*-LA. The best known is the antitumoral activity observed for the complex of bovine *α*-lactalbumin and oleic acid (BAMLET), the bovine counterpart of HAMLET (human *α*-lactalbumin and oleic acid), that seems to kill tumor cells via a mechanism involving lysosomal membrane permeabilization. It consists of the calcium depleted apo form of *α*-LA in the aforementioned molten globule state, which is stabilized by a fatty acid cofactor. It is noteworthy that the *α*-LA/fatty acid interaction is stereo-specific; it is for this reason that unsaturated *cis*-fatty acids bind to *α*-LA, and only the C18 : 1 : 9 *cis*-fatty acid (oleic acid), that interacts with *α*-LA in a compact conformation, is active against tumor cells [21, 22]. BAMLET accumulates rapidly and specifically in the endolysosomal compartment of tumor cells and induces an early leakage of lysosomal cathepsins into the cytosol followed by the activation of the protein Bax, a proapoptotic Bcl-2 family protein. 

*α*-LA can also be a potent calcium concentration-elevating and apoptosis-inducing agent [23]. Multimeric form of *α*-LA was shown to promote apoptosis in transformed and immature cells while sparing mature epithelial cells. During this process calcium levels are elevated, allowing a connection between calcium levels and apoptosis. Probably, *α*-LA interacts with cell surface modulators altering calcium transport rates, intracellular calcium, and cell growth rate. High affinity to metal ions is mainly due to junction to subdomains at 79–88 containing five aspartates [24]. Furthermore, this protein possesses antiproliferative effects on colon adenocarcinoma cell lines (CaCo2 or HT-29 monolayers). Low concentrations of *α*-LA (10–25 *µ*g/mL) stimulate growth during the first 3 to 4 days. After growing for 4 days, proliferation ceases and viable cell numbers decrease dramatically, suggesting a delayed initiation of apoptosis [25]. 

### 3.2. *β*-Lactoglobulin

*β*-Lactoglobulin (*β*-Lg) is quantitatively a noncasein protein in bovine milk (58% w/w). It is a small, soluble, and globular protein, but its quaternary structure depends on the medium pH. At pH of 3.0 and above 8.0, *β*-Lg is a monomer molecule with a molecular weight of 18 kDa, while, at pH between 7.0 and 5.2, it is a stable dimer with molecular mass of about 36.7 kDa; at pH between 5.2 and 3.5 it is an octamer with molecular mass of 140 kDa. *β*-Lg is composed mainly of *β*-sheet motifs and consists of 162 aminoacid residues [5].

*β*-Lg has been implicated in providing protection against development of cancer in animal models when delivered orally. The mechanism of anticancer activity of *β*-Lg may be related to its sulphur aminoacid content. This suggests a possible role in protecting DNA in methylated form. Indeed, the aminoacid composition of *β*-Lg plays an important role in preventing oxidative damage. Particularly, *β*-Lg influences tissue levels of the thiol-glutathione, a multifunctional tripeptide, that binds and eliminates endogenous and exogenous mutagens and carcinogens.

Since the precursors for the synthesis of glutathione, as cysteine and glutamylcysteine, are provided by *β*-Lg, it is possible to establish a relationship between tripeptide levels and *β*-Lg [26]. The high nutritional and functional value of *β*-Lg is widely recognized and has made this protein an ingredient of choice in the formulation of modern foods and beverages [27].

### 3.3. Bovine Serum Albumin

Bovine serum albumin (BSA) is not synthesized in the mammary gland, but appears in milk following passive leakage from the blood stream. It contains 582 aminoacid residues with a molecular weight of 66,267 kDa; it also possesses 17 intermolecular disulphide bridges and one thiol-group at residue 34 [19]. Because of its size, BSA can bind to free fatty acids and other lipids as well as flavour compounds [28]—a feature that is severely hampered upon denaturation. Its heat-induced gelation at pH 6.5 is initiated by an intermolecular thiol-disulphide interchange, similar to what happens with *β*-Lg [29].

BSA inhibits the growth of MCF-7 human breast cancer cell line [30]. Inhibition of MCF-7 cell proliferation by BSA is in a concentration-dependent manner. BSA may affect cell proliferation by modulating the activities of autocrine growth regulatory factors.

BSA has also been reported to exhibit strong antiproliferative effects against a Chinese hamster epithelial cell line, although the mechanisms for this inhibition remain unclear [31].

### 3.4. Lactoferricin

Lactoferrin, an iron-binding glycoprotein with a molecular weight of about 80 kDa (703-amino acid), is mainly found in external secretions that include breast milk and saliva and in the secretory granules of neutrophils. In addition to its antimicrobial effects, it is well known to possess a variety of biological activities, like regulation of immune response [32, 33], cells transcriptional activation [34], and antiviral activity [35]. The antimicrobial activity of bovine lactoferrin (LF-B) has been attributed to the bovine lactoferricin fragment (LfcinB), which, unlike the parental glycoprotein, displays no iron-binding capacity. In fact, the LfcinB is considered as the active domain responsible for antimicrobial activity of LF-B, against a wide range of microorganisms.

Lactoferricin is a cationic peptide produced by acid-pepsin hydrolysis of mammalian lactoferrin [36], and it consists of 25 aminoacid residues (FKCRRWQWRMKKLGAPSITCVRRAF), including two cysteine residues that create a disulfide bond linking the highly positively charged NH_2_-terminal region and the COOH-terminal region of the peptide [37]. LfcinB has a high content of asymmetrically clustered basic aminoacid residues, giving the peptide a net positive charge of 7.84 at pH 7.0.

Its amphipathic property is given by the twisted *β*-sheet structure that LfcinB assumes in aqueous solution, in which nearly all of the hydrophobic residues are found on one face with the basic aminoacid residues positively charged on the opposing face.

The cytotoxic activity of LfcinB has been demonstrated *in vitro* on many different types of rat and human cancer cell lines, including leukemia, fibrosarcoma, various carcinoma, and neuroblastoma cells [38–41], at concentrations that do not substantially affect the viability of normal fibroblasts, lymphocytes, epithelial cells, endothelial cells, or erythrocytes [40, 42]. The selectivity of action of LfcinB is due to its strongly cationic nature that allows the peptide to target negatively charged cancer cells, whereas healthy untransformed cells are spared because of their net neutral charge due to the high content of zwitterionic phosphatidylcholine in the outer membrane leaflet [43]. The net negative charge that is associated with the outer membrane leaflet of many cancer cells results from differential branching and sialic acid content of N-linked glycans associated with transmembrane glycoproteins [44], as well as elevated expression of anionic molecules such as phosphatidylserine [45, 46] and O-glycosylated mucins [43, 47].

It is this selectivity of action that makes LfcinB unable to bind to PC3 prostate carcinoma cells. Therefore, it seems possible that some cancer cells may be refractory to the cytotoxic effect of LfcinB treatment due to an insufficient net negative charge to promote a strong electrostatic interaction with cationic LfcinB.

Since the cytotoxic activity of LfcinB against cancer cells strongly depends on its structure, amphipathic nature and high net positive charge (+7, if compared to +4 for antimicrobial activity), this activity is, therefore, increased in LfcinB derivatives with clear cationic and hydrophobic moieties, while a glutamic acid-containing homologue of murine lactoferricin lacks the ability to kill cancer cells [48–50].

The activities against fibrosarcoma and neuroblastoma rat cells instead of human cells can be explained by a mechanism that induces the formation of transmembrane pores that allow the peptide to enter the cytoplasmic compartment of the cancer cell and colocalize with negatively charged mitochondria, causing cell death primarily via necrosis by a cell membrane lytic effect. In fact in terms of structural membrane changes, insertion of LfcinB [51] promotes the formation of inverted hexagonal or bicontinuous cubic phases in membrane mimetic systems [52–56]. In contrast, LfcinB kills human T-leukemia and breast cancer cells by triggering caspase-3 activation through the mitochondrial pathway of apoptosis.

According to studies conducted by Yoo et al. [38], LfcinB is able to kill THP-1 human monocytic leukemia cells by the activation of apoptotic pathways. Its apoptosis-inducing activity is associated with the production of intracellular ROS and activation of Ca^2+^/Mg^2+^-dependent endonucleases. Treatment of THP-1 cells with LfcinB (100 **μ**g/mL) elicited apoptosis with maximal activity after about 10 h, whereas LF-B did not induce cell death even at a dose of 500 **μ**g/mL. THP-1 cells treated with LfcinB exhibited fragmented DNA in a dose-dependent manner, a time- and dose-dependent progressive reduction in cell membrane integrity that allowed LfcinB to enter the cytoplasmic compartment. However, the membrane-lytic effect of extracellular LfcinB, that does not depend on internalization of the peptide, may also contribute to the LfcinB-induced cytotoxicity. In fact, the addition of Zn^2+^, an inhibitor of endonucleases, which requires divalent cations for their full activity, inhibited LfcinB-induced cell death. However, LfcinB-induced apoptosis in THP-1 cells was effectively abolished by the addition of antioxidants, such as NAC (N-acetyl-cysteine) and GSH (glutathione), similarly to that induced by H_2_O_2_.

The capacity of LfcinB to induce apoptosis in cancer cells through a pathway mediated by the production of the intracellular ROS and activation of Ca^2+^/Mg^2+^-dependent endonucleases was confirmed by Mader et al. [57], who also documented the mitochondrial pathway of apoptosis for LfcinB. Mader et al. [57] demonstrated that LfcinB-induced apoptosis in human T-leukemia cells was triggered by a sequence of events consisting of LfcinB-mediated permeabilization of the cell membrane, uptake across the damaged cell membrane, colocalization with mitochondria, and depolarization of mitochondria, resulting in cytochrome C release, combination of cytosolic Apaf-1 responsible for recruiting and activating procaspase-9, thereby forming the apoptosome that triggers the caspase cascade and leads to cell death by apoptosis.

Other studies have reported that induction of apoptotic or necrotic cell death is dependent on the concentration of the peptide [41, 58] because the cytotoxic activity of LfcinB is reduced in the presence of high concentrations of serum. In fact, systemic or intratumoral administration of LfcinB inhibits the *in vivo* growth and/or metastasis of several different tumor types in mice [38, 39, 41]. This inhibitory effect of Lfcin-induced apoptosis is the result of neutralization by anionic serum components rather than proteolytic degradation.

It has been recently shown that LfcinB-induced apoptosis in B-lymphoma cells does not involve the caspase cascade but determines apoptosis via the activation of cathepsin B [59].

Mader et al. [60] have shown that LfcinB may interfere with the interaction of the heparin-binding growth factors bFGF and VEGF with their receptors on the surface of endothelial cells, resulting in decreased endothelial cell proliferation and diminished angiogenesis [61]. Although the exact mechanism by which LfcinB interacts with heparin-like molecules has not been elucidated yet, it was hypothesized that the affinity that LfcinB displays for heparin-like structures is the result of electrostatic interactions between the positive charge of LfcinB and negative charge of heparin and heparan sulfate. This antiangiogenic activity is dependent on the primary structure of the peptide since a scrambled peptide comprised of the same aminoacid residues fails to effectively compete with bFGF or VEGF for heparin-like binding sites on endothelial cells.

However, the main limitation of systemic administration of LfcinB for the antiangiogenic therapy is the susceptibility of the peptide to enzymatic digestion and inactivation through interactions with anionic serum components.

## 4. Conclusions 

Peptides derived from milk protein have been shown to exert beneficial effects on human health. These biological properties may play an important role in the development of medical foods that treat or mitigate the effects of diseases. Bioactive peptide preparations have the potential to be used in the formulation of functional foods and cosmetics and as potent drugs having well-defined pharmacological effects. With the rise of consumer concerns about the deleterious effects of chemical preservatives and the increasing preference for natural components, milk-derived bioactive substances may have value in food preservation and nutraceuticals. Application of enrichment protocols such as membrane processing and chromatographic isolation may also be an area of future interest in the extraction of potent biofunctional peptides from milk and dairy products and their subsequent utilization as functional food ingredients. Molecular studies are required to clarify the mechanisms by which the bioactive peptides exert their activities.

## Figures and Tables

**Table 1:** Anticancer peptide and proteins from bovine milk [5].

Family proteins	Protein precursors		Concentration (g/L)	M.W.^†^	Peptide fragments	Amino acid sequence
			24–28		Caseinphosphopeptides	PPPEE^*ζ*^
	*α*_ s1_-casein	*α*_ s1_-CN	12–15	22.1–23.7	*α*_ s1_-casein f(90–95)	RYLGYL
					*α*_ s1_-casein f(90–96)	RYLGYLE
Caseins					*α*_ s1_-casomorphin f(158–162)	YVPFP
	*β*-casein	*β*-CN	9–11	23.9–24.1	*β*-Casomorphins 5 f(60–64)	YPFPG
					*β*-Casomorphins 7 f(60–66)	YPFPGPI
					Morphiceptin f(60–63)-NH_2_	YPFP-NH_2_

			5–7			
	*β*-lactoglobulin	*β*-lg	2–4	18.3		
Whey proteins	*α*-lactalbumin	*α*-la	1–1.5	14.2		
	Bovine serum albumin	BSA	0.1–0.4	66		
	Lactoferrin	Lf	0.1	80	Bovine lactoferricin (LfcinB)	FKCRRWQWRMKK LGAPSITCVRRAF

^*ζ*^Characteristic cluster sequence of CPPs.

^†^Molecular weight expressed in kDa.
